# Elevated Blood Pressure in Adolescence Is Attributable to a Combination of Elevated Cardiac Output and Total Peripheral Resistance: Evidence Against a Hyperkinetic State

**DOI:** 10.1161/HYPERTENSIONAHA.118.11925

**Published:** 2018-10-01

**Authors:** Chloe Park, Abigail Fraser, Laura D. Howe, Siana Jones, George Davey Smith, Debbie A. Lawlor, Nish Chaturvedi, Alun D. Hughes

**Affiliations:** 1 From the Department of Population Science and Experimental Medicine, Institute of Cardiovascular Sciences, University College London, United Kingdom (C.P., S.J., N.C., A.D.H.); 2 Population Health Sciences, Bristol Medical School, University of Bristol, United Kingdom (A.F., L.D.H., G.D.S., D.A.L.); 3 MRC Integrative Epidemiology Unit at the University of Bristol, United Kingdom (A.F., L.D.H., G.D.S., D.A.L.); 4 MRC Unit for Lifelong Health and Ageing at UCL, London, United Kingdom (N.C., A.D.H.).

**Keywords:** blood pressure, cardiac output, heart rate, stroke volume, vascular resistance

## Abstract

Unlike in older people, it has been suggested that elevated blood pressure (BP) in young people is because of high cardiac output accompanied by normal total peripheral resistance (TPR)—a hyperkinetic/hyperdynamic circulation. We investigated this in a large, United Kingdom-based birth cohort of adolescents. The study was conducted on 2091 17-year-old participants in the ALSPAC (Avon Longitudinal Study of Parents and Children)—a prospective population-based birth cohort study. BP measurement and echocardiography were performed, and heart rate (HR), stroke volume (SV), and TPR were calculated. Data are means (SD). Higher quintiles of systolic BP were associated with higher SV, higher HR, and higher TPR. The proportional contribution made by SV, HR, and TPR to mean arterial pressure differed little by systolic BP quintile (SV [32%–34%], HR [25%–29%], and TPR [39%–41%]). Higher BP is attributable to a combination of higher cardiac output (ie, SV×HR) and higher TPR in a population-based sample of adolescents. There is no evidence of a disproportionate contribution from elevated cardiac output at higher BP levels.

## Abstract 2

Supplemental Digital Content is available in the text.

**See Editorial Commentary, pp 1093–1094**

Elevated blood pressure (BP) accounts for two thirds of all strokes and a half of all coronary heart diseases, and the relationship between BP and risk is continuous and log-linear across the range of BP.^1^ BP tracks from adolescence into adulthood,^2,3^ and BP measured in adolescence is a predictor of cardiovascular events and renal disease ≤50 years later.^4–6^ Elevated BP is associated with increased left ventricular (LV) mass and LV hypertrophy in adults^7^ and children.^8^ Increased LV mass and hypertrophy are associated with subsequent cardiovascular risk, independent of other risk factors, including BP.^9–14^ Mean arterial pressure (MAP) in excess of right atrial pressure is the driving force for flow in the systemic circulation and is determined by cardiac output (CO; calculated as stroke volume [SV]×heart rate [HR])×total peripheral resistance (TPR) of the systemic circulation. Several previous studies^15–17^ have suggested that high BP in young people may be explained by elevated CO rather than elevated TPR, whereas the latter is more typical in older people with established hypertension.^17^ This high CO state has been termed a hyperkinetic (or hyperdynamic) circulation.^18^ This has been proposed to be a precursor to adult hypertension, with a transition from a high CO-normal TPR to a normal CO-high TPR state with aging.^19^ However, not all studies have confirmed these observations^20,21^ and those that have observed hyperkinetic hemodynamics have often been based on small sample sizes or have studied selected individuals (eg, those with borderline hypertension), which may bias relationships or render them nongeneralizable.

We, therefore, aimed to investigate whether higher BP in young people is explained by a hyperkinetic state and whether elevated HR and SV made a disproportionate contribution to elevated BP in a large, population-based cohort of adolescents.

## Methods

The ALSPAC (Avon Longitudinal Study of Parents and Children) is a prospective population-based birth cohort study that recruited 14 541 pregnant women residents in Avon, United Kingdom, with expected dates of delivery between April 1, 1991, and December 31, 1992 (http://www.alspac.bris.ac.uk).^22^ Since 7 years of age, surviving participants have been invited to regular follow-up clinics. The study website contains details of all the data that are available through a fully searchable data dictionary (http://www.bris.ac.uk/alspac/researchers/data-access/data-dictionary/) and also includes details of representativeness of the sample. Five thousand two hundred seventeen participants attended the clinic assessment at 17 years of age. Because of limited time and equipment availability, 1 in 2 of daily clinic attenders were invited to undergo echocardiography. This study is based on these individuals. Ethical approval was obtained from the ALSPAC Law and Ethics Committee and the Local Research Ethics Committee. Participants provided written informed consent.

### Measurement of Peripheral BP

Sitting peripheral systolic BP (SBP), diastolic BP, and HR were measured using an Omron 705 IT oscillometric BP monitor. Arm circumference was measured, and an appropriate cuff size was chosen according to manufacturer’s instructions. The average of the final 2 of 3 readings was used in the analysis. Pulse pressure was calculated as the difference between SBP and diastolic pressures. MAP was calculated as diastolic BP+1/3 (SBP−diastolic BP).

### Measurement of Cardiac Structure and Function

Echocardiography was performed using a HDI 5000 ultrasound machine (Phillips) equipped with a P4-2 Phased Array Ultrasound Transducer by 1 of 2 echocardiographers using a standard examination protocol. All measurements and calculations were made according to American Society of Echocardiography guidelines.^23^ LV mass was indexed (LVMI) to height^2.7^ and TPR was calculated as MAP/CO. Quality control was performed throughout the study, and reproducibility of echocardiographic measurement was assessed by recalling 30 participants and repeating their measurements. The intraclass correlation of repeated echocardiographic measurements was excellent: 0.75 to 0.93 (intraobserver) and 0.78 to 0.93 (interobserver).

### Other Measures

Age at clinic assessment and sex were recorded. Other demographic and lifestyle data were ascertained from questionnaire data; socioeconomic position was assigned by paternal occupation, education was classified as in full-time education or not, alcohol consumption was assessed as the number of drinks containing alcohol consumed on a typical day, smoking was categorized as never, ever but not current, or current. Weight and height were measured while the subjects were wearing light clothing and no shoes. Weight was measured to the nearest 0.1 kg by using scales (Tanita Europe BV, Amsterdam, the Netherlands). Height was measured to the nearest 0.1 cm by using a Harpenden stadiometer (Holtain, Ltd, Crymych, United Kingdom). Body mass index (BMI) was calculated as weight (kg)/height^2^ (m^2^).

### Data and Statistical Analysis

Individuals with diabetes mellitus (n=21), familial hypercholesterolemia (n=7), and known heart disease (n=3) or women who were pregnant (n=15) were excluded. Data from a further 25 individuals without information on sex or BP were also not used. MAP and LVMI were subdivided into quintiles to examine associations with SV, HR, and TPR without assuming linear relationships across the entire range of variables. The primary analysis was performed on both sexes combined, but we also examined both sexes separately. MAP was used for these analyses because on a priori grounds, HR and TPR would not be expected to contribute to pulse pressure, but a further sensitivity analysis was performed using quintiles of SBP because this is more widely clinically used as a measure of BP than MAP. Associations between LVMI or relative wall thickness (RWT) and SV, HR, and TPR were examined by multiple linear regression with age, sex, height, BMI, socioeconomic position, education status, alcohol consumption, and smoking as potential confounders; both LVMI and BMI are indexed to height, which could introduce some colinearity between these measures, so we also constructed models in which weight was substituted for BMI.

Descriptive statistics for continuous variables are presented as means (SD) or medians (interquartile ranges) for skewed data. The primary analysis was performed as a complete-case analysis; however, to check that missingness did not influence results, multiple imputation was also used to impute missing outcome or covariable data for participants who met the inclusion criteria. The imputation equations included all outcomes, exposures, and covariables, and 20 imputed datasets were created. Because imputation had minimal effects on results, these results are not presented.

## Results

### Characteristics of Study Participants

Characteristics of those included in analyses are shown in Table 1. As expected, women were shorter, weighed less, and had lower SBP and higher HR than men; 64 individuals (3%; 6% men and 1% women) had a clinic BP ≥140/90 mm Hg. Compared with those not studied, participants were slightly older, more likely to be in current full-time education, less likely to be smokers, and had slightly higher resting HR (Table S1 in the online-only Data Supplement). Characteristics of the individuals allocated to quintiles of MAP are shown in Table S2.

**Table 1.**
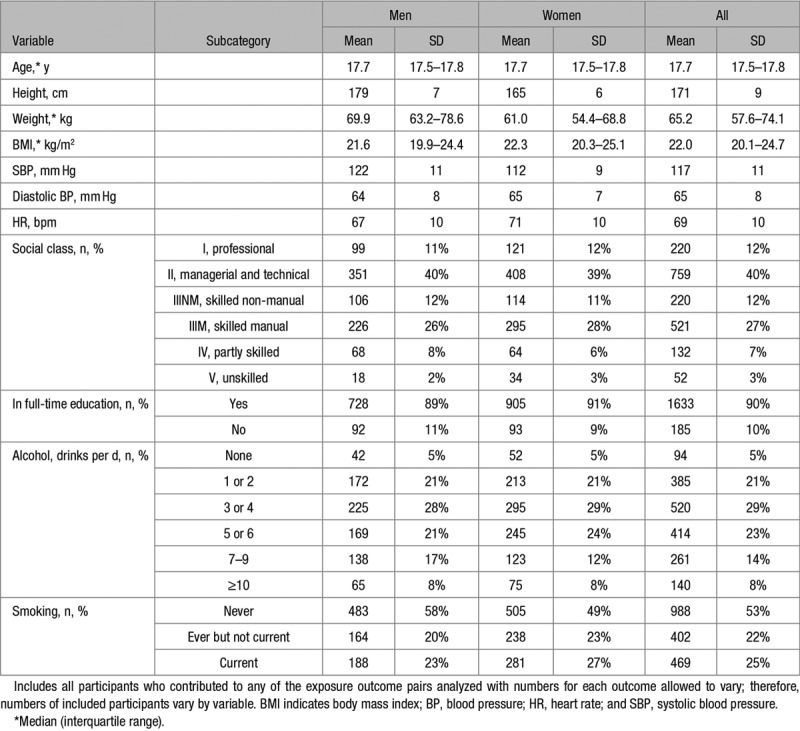
Characteristics of Participants Who Attended the 17-y Clinic Assessment and Were Included in Analyses

**Figure 1.**
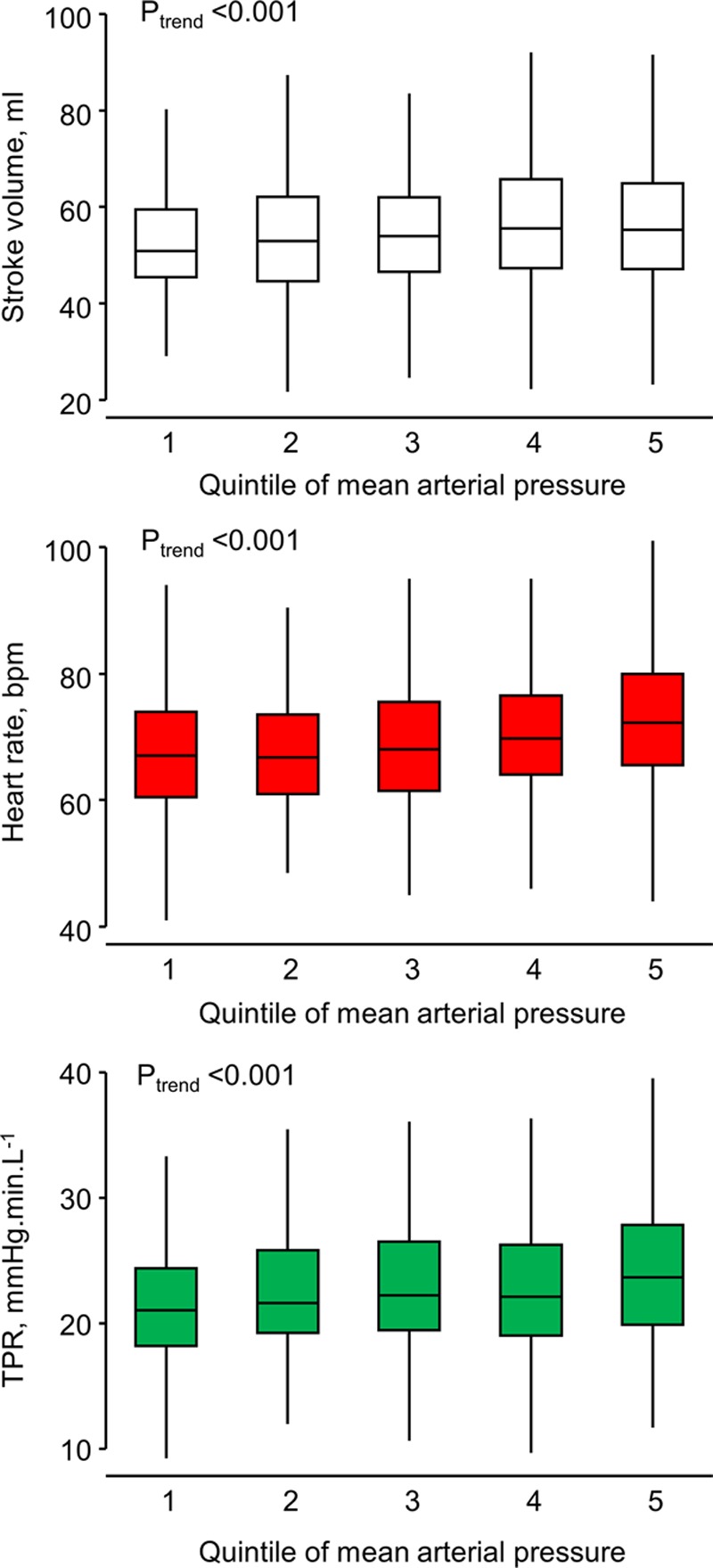
Box and whisker plots of (**A**) stroke volume (white), (**B**) heart rate, (red) and (**C**) total peripheral resistance (TPR; green) by quintiles of mean blood pressure.

The values of SV, HR, CO, and TPR in each quintile of MAP are shown in Table 2. Individuals in higher quintiles of MAP had higher HR and higher TPR, but the relationships between MAP and SV were weak in both sexes (Figure 1A through 1C). As would be expected from the HR and SV results, there was a positive graded relationship between MAP and CO (β-coefficient for linear trend, 0.11 [0.08–0.14]; *P*<0.001). There was no evidence of modification by sex for any of the findings; analyses stratified by sex are shown in Figure S1.

**Table 2.**
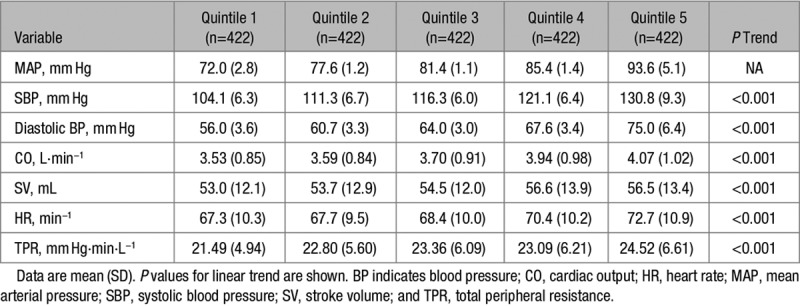
Hemodynamics by Quintile of MAP

**Figure 2.**
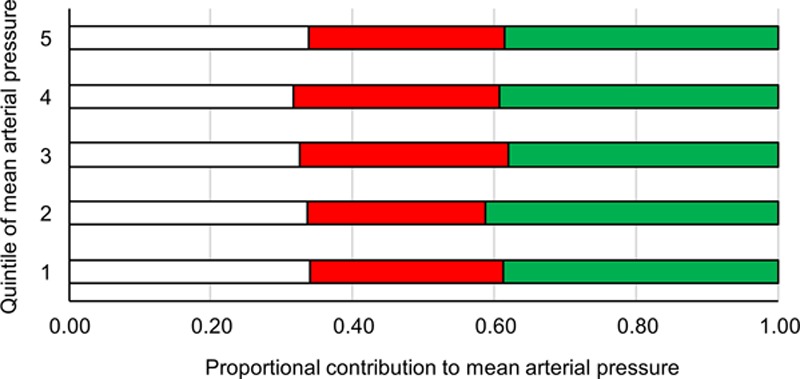
Proportionate contribution of stroke volume (white), heart rate (red), and total peripheral resistance (green) to mean arterial pressure in each quintile of systolic blood pressure.

The proportional contribution made by SV, HR, and TPR to MAP hardly differed across MAP quintiles (Figure 2), and the contribution made by CO compared with TPR also remained constant across quintiles of MAP, with both rising almost in parallel with increasing quintile of MAP.

In a multiple regression model adjusted for potential confounders, higher SV and TPR were associated with higher LVMI (Table 3), whereas higher HR and TPR but lower SV were associated with higher RWT (Table 3). When SV was replaced by end-diastolic volume in these models, there was a similarly strong association between end-diastolic volume and LVMI and RWT (data not shown). Replacement of BMI with weight in models also had negligible effects on findings (data not shown).

**Table 3.**
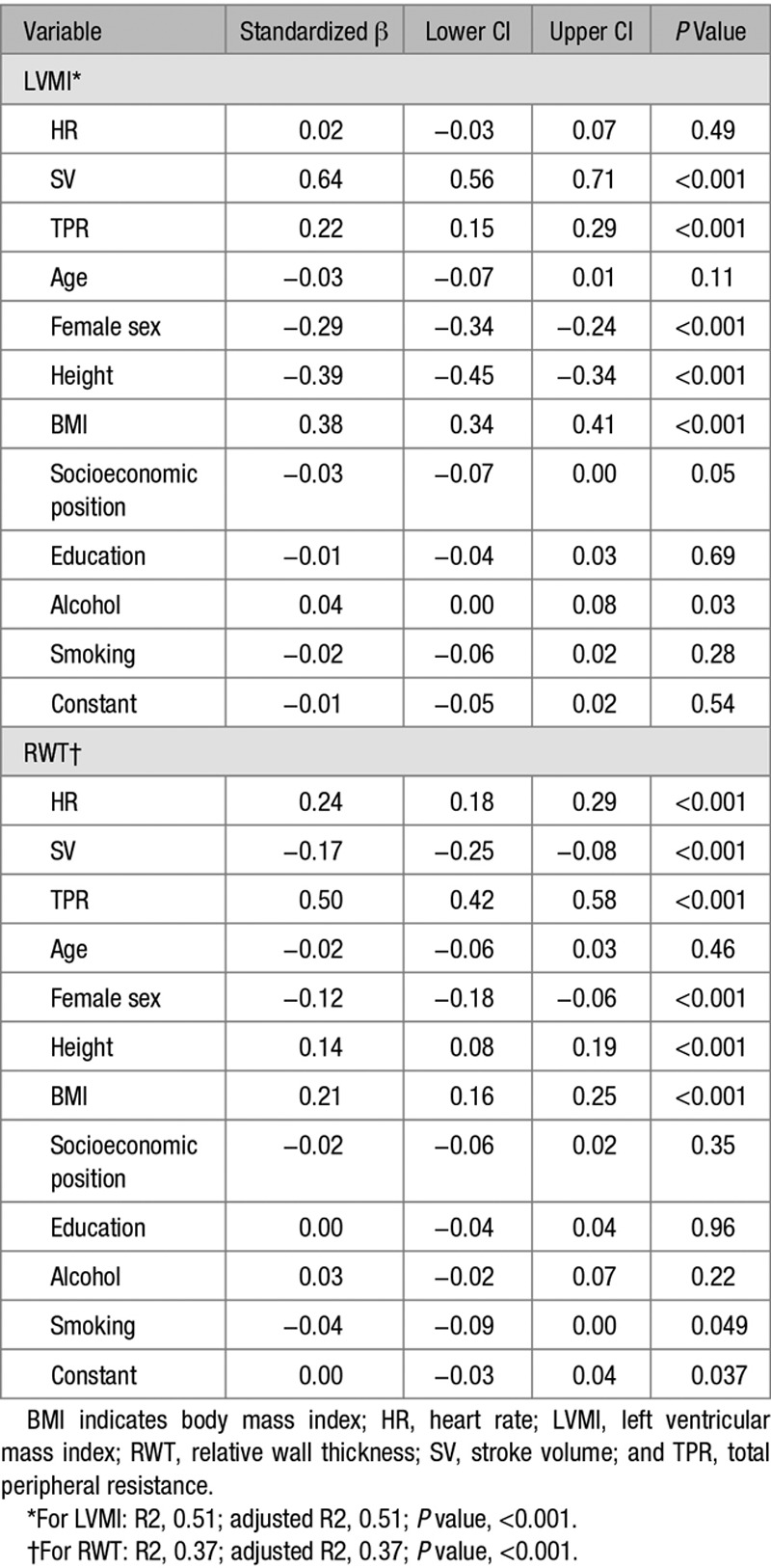
Multilinear Regression Model of Association Between SV, HR, and TPR and LVMI and RWT, Adjusted for Potential Confounders

## Discussion

Higher BP in adolescence is because of a combination of higher HR, SV, and TPR as is commonly observed in adults.^24^ Our data provide no evidence that elevated HR, SV, or their product CO (ie, SV×HR) makes the sole or even predominant contribution to elevated BP at this age. Greater LVMI was associated with higher SV and TPR but not HR, suggesting that the greater wall mass may largely represent an adaptation to increased work related to preload and afterload. There was no evidence of interactions by sex indicating that these findings apply to both men and women.

Our observations do not lend support to the idea of a specific hyperkinetic or hyperdynamic state in young people with high BP as proposed previously.^15–17^ Reasons for these differences between studies are uncertain: possibly sample categorization and selection contribute to the observed differences. Julius et al^16^ subdivided hypertensive individuals in Tecumseh into normokinetic and hyperkinetic subgroups, both groups had elevated TPR, although only the hyperkinetic group had an elevated CO—it is noteworthy that the normokinetic group predominated by almost 2:1 (62 compared with 37). Lund Johanssen^17^ observed higher CO in 19 hypertensive individuals compared with 11 normotensive individuals aged 17 to 29 years, a comparable difference was not seen in older people; however, TPR was higher on average in hypertensive individuals at each age group studied. In the ENIGMA study of university students in the United Kingdom,^25^ young people with essential hypertension had elevated TPR, higher HR, and lower SV (and consequently a similar CO) to normotensive individuals, whereas young people with isolated systolic hypertension had higher MAP and SV, but HR and TPR did not differ from normotensive individuals. In ENIGMA, the normotensive, essential hypertensive, and isolated systolic hypertensive groups differed in terms of height, sex, and BMI, and it is likely that these differences may have contributed to the different hemodynamic patterns observed.

Our observations relating HR, SV, and TPR to LVMI and RWT are consistent with previous studies in older adults^26,27^ and suggest that LV adaptation to hemodynamic load is similar in adolescence to that observed in later life. The strong association between SV (or end-diastolic volume) on LVMI and RWT is also in keeping with previous findings in adults^26,27^ and is likely to reflect the strong influence of volume load on chamber size, wall stress, and LV hypertrophy.^28^

Our study has strengths and limitations. ALSPAC is an unselected general population study that is reasonably representative of the contemporary UK population.^29^ As is expected in adolescence,^4–6^ the prevalence of hypertension was low; therefore, we cannot exclude that selected hypertensive adolescents may show different hemodynamic characteristics. All participants were of similar age when studied minimizing the influence of age on our findings but limiting the generalizability of our findings to other ages. The ALSPAC cohort is predominantly of white European origin, and it should not be assumed that our results will apply to other populations.

Although nested within a cohort, this study was cross-sectional, and, therefore, the question of whether people with high CO and normal TPR are at higher risk of essential hypertension or whether there are age-dependent differences in the trajectories of the hemodynamic determinants of MAP cannot be addressed. However, in the Framingham study^30^ after adjustment for age and baseline BP, no hemodynamic variables were significantly associated with the incidence of hypertension during a 4-year follow-up period, and in a study of young Swedish men, there was no significant correlation between baseline CO and BP measured 30 years later.^31^

## Perspectives

Higher BP in adolescents is attributable to a combination of higher SV, higher HR, and higher TPR. Relationships between measures of hemodynamic load and LV mass and RWT were qualitatively similar to those reported previously in older adults. There is no evidence of a disproportionate contribution from elevated HR or SV that explains higher BP levels in youth.

## Acknowledgments

We are extremely grateful to all the families who took part in this study, the midwives for their help in recruiting them, and the whole ALSPAC (Avon Longitudinal Study of Parents and Children) team, which includes interviewers, computer and laboratory technicians, clerical workers, research scientists, volunteers, managers, receptionists, and nurses. C. Park and A.D. Hughes had full access to the data and took responsibility for the integrity of the data and its analysis.

## Sources of Funding

The Medical Research Council (MRC), the Wellcome Trust (092731), and the University of Bristol provide core funding support for ALSPAC (Avon Longitudinal Study of Parents and Children). A. Fraser, L.D. Howe, G. Davey Smith, D.A. Lawlor, N. Chaturvedi, and A.D. Hughes work in units that receive funding from the UK MRC (MC_UU_12013/1, MC_UU_12013/5, MC_UU_12013/9, and MC_UU_12019/1). A. Fraser and L.D. Howe are funded by UK MRC fellowships (MR/M020894/1 and MR/M009351/1, respectively). Additional funding for this study came from the Wellcome Trust (086676/7/08/Z) and the British Heart Foundation (PG/06/145 and CS/15/6/31468). The views expressed in this article are those of the authors and not necessarily those of any funding body or others whose support is acknowledged. The funders had no role in study design, data collection and analysis, decision to publish, or preparation of the manuscript.

## Disclosures

None.

## Supplementary Material

## Novelty and Significance

### What Is New?

- Evidence that high blood pressure (BP) in adolescence is because of a hyperkinetic/hyperdynamic circulation is conflicting, and many previous studies have been based on small samples or selected individuals. We investigated this question in a large, population-based cohort of adolescents. We found that higher BP is attributable to a combination of higher cardiac output (ie, stroke volume×heart rate) and higher total peripheral resistance in a population-based sample of adolescents. There is no evidence of a disproportionate contribution from elevated cardiac output at higher BP levels.

### What Is Relevant?

- This study provides new insights into the hemodynamics of high BP in young people.

### Summary

Elevated BP in adolescents is attributable to a combination of higher stroke volume, higher heart rate, and higher total peripheral resistance. Relationships between measures of hemodynamic load and left ventricular mass and relative wall thickness were qualitatively similar to those reported previously in older adults. There is no evidence of a disproportionate contribution from elevated heart rate and stroke volume that explains higher BP levels in youth.
