# Supplementary material for: Elevated Blood Pressure in Adolescence Is Attributable to a Combination of Elevated Cardiac Output and Total Peripheral Resistance: Evidence Against a Hyperkinetic State
Source: Hypertension. 2018 Oct 1;72(5):1103–8. doi: 10.1161/HYPERTENSIONAHA.118.11925 (PMC6181289; doi:10.1161/HYPERTENSIONAHA.118.11925)
Supplement: Supplementary file 1 [file hyp-72-1103-s001.pdf]

Supplemental Material

Elevated blood pressure in adolescence is attributable to a combination of elevated cardiac output and total peripheral resistance: evidence against a hyperkinetic state

Chloe Park,[1] BSc, PhD, Abigail Fraser[2, 3], MA, MPH, PhD, Laura D. Howe [2,3], MSc, PhD, Siana Jones[1], MSc, George Davey Smith[2,3], MA, MD, BChir, MSc, Debbie A. Lawlor[2,3], MBChB, MSc, PhD, Nish Chaturvedi[1,4], MB, BS, MSc, MD, MRCP, Alun D. Hughes[1,4], MB, BS, PhD, FBPharmacolS.

1. Institute of Cardiovascular Sciences, UCL, London, UK
2. Population Health Sciences, Bristol Medical School, University of Bristol, Bristol, UK.
3. MRC Integrative Epidemiology Unit at the University of Bristol, Bristol, UK.
4. MRC Unit for Lifelong Health & Ageing at UCL, London, UK

Corresponding author:

Alun Hughes,

Department of Population Science & Experimental Medicine,

Institute of Cardiovascular Sciences,

University College London, London, WC1E 6BT

Phone: +44 20 7679 9432

Fax: +44 20 7580 1501

E-mail: alun.hughes@ucl.ac.uk

Table S1. Comparison of attendees included or not included in study

Variable	Attendees not included N	Attendees not included Mean / Median / (%)	Attendees not included SD/IQR	Attendees included N	Attendees included Mean/ Median/(%)	Attendees included SD/IQR	P
Age[a], y	2943	17.8	(0.5)	2091	17.7	(0.3)	<0.001
Male sex, N (%)	1273	(25.2)		939	(18.6)		0.2
Height, cm	2855	171.1	(9.3)	2057	171.2	(9.4)	0.6
Weight[a], kg	2852	64.4	(16.3)	2060	65.2	(16.5)	0.9
Body mass index[a], kg/m^2	2851	21.9	(4.4)	2056	22.0	(4.7)	>0.9
Systolic BP, mmHg	2960	116.3	(11.4)	2091	116.6	(11.3)	0.4
Diastolic BP, mmHg	2960	64.7	(7.7)	2091	64.6	(7.5)	0.8
Heart rate, bpm	2960	70.6	(10.8)	2091	69.3	(10.4)	<0.001
Socioeconomic status, N (%)							0.2
I - Professional	274	(6.0)		220	(4.9)		
II - Managerial and technical	974	(21.5)		759	(16.7)		
IIINM - Skilled non-manual	319	(7.0)		220	(4.9)		
IIIM - Skilled manual	790	(17.4)		521	(11.5)		
IV - Partly skilled	200	(4.4)		132	(2.9)		
V - Unskilled	71	(1.6)		52	(1.1)		
In education, N (%)	1894	(47.3)		1633	(40.8)		0.003
Alcoholic drinks per day, N (%)							0.06
None	115	(5)		94	(5)		
1 or 2	437	(11.5)		385	(10.2)		
3 or 4	560	(14.8)		520	(13.7)		
5 or 6	568	(15.0)		414	(10.9)		
7 to 9	316	(8.3)		261	(6.9)		
10 or more	189	(5.0)		140	(3.7)		
Smoking, N (%)							<0.001
Never	1023	(24.9)		988	(24.1)		
Ever but not current	550	(13.4)		402	(9.8)		
Current	673	(16.4)		469	(11.4)		

[a] median (interquartile range). Discrepancies in N in some variables are attributable to missing data. Abbreviations BP, blood pressure; bpm, beats per minute.

Table S2. Characteristics of the individuals allocated to quintiles of MAP

	Quintile 1		Quintile 2		Quintile 3		Quintile 4		Quintile 5	
**Variable**	**Mean / N**	**SD / (%)**	**Mean / N**	**SD / %**	**Mean / N**	**SD / %**	**Mean / N**	**SD / %**	**Mean / N**	**SD / %**
Age, y	17.7	0.3	17.7	0.3	17.7	0.3	17.7	0.3	17.7	0.3
Male sex, N (%)	126	(30)	156	(37)	191	(46)	231	(53)	235	(59)
Height, cm	167.9	8.3	169.8	9.0	171.9	9.3	172.4	9.1	174.0	9.9
Weight, kg	59.1	11.1	63.3	13.4	66.6	15.2	67.6	16.6	72.0	18.9
Body mass index, kg/m^2	21.0	3.5	21.7	3.7	22.0	4.3	22.5	4.8	23.6	7.0
Systolic BP, mmHg	104.1	6.3	111.3	6.7	116.3	6.0	121.1	6.4	130.8	9.3
Diastolic BP, mmHg	56.0	3.6	60.7	3.3	64.0	3.0	67.6	3.4	75.0	6.4
Heart rate, bpm	67.3	10.3	67.7	9.5	68.4	10.0	70.4	10.2	72.7	10.9
Socioeconomic position, N (%)										
I - Professional	52	(14)	47	(12)	47	(12)	44	(11)	30	(8)
II - Managerial and	153	(40)	143	(37)	141	(37)	171	(44)	151	(42)
technical	44	(12)	47	(12)	56	(15)	35	(9)	38	(10)
IIINM - Skilled	111	(29)	111	(29)	92	(24)	106	(27)	101	(28)
non-manual	17	(4)	26	(7)	30	(8)	27	(7)	32	(9)
IIIM - Skilled	4	(1)	14	(4)	13	(3)	10	(3)	11	(3)
In Education, N (%)	329	(90)	319	(89)	336	(91)	339	(89)	310	(90)
Alcoholic drinks per day, N (%)										
None	23	(6)	21	(6)	18	(5)	20	(5)	12	(3)
1 or 2	66	(18)	59	(17)	86	(23)	87	(23)	87	(25)
3 or 4	131	(36)	94	(27)	113	(30)	95	(25)	87	(25)
5 or 6	82	(23)	96	(27)	76	(20)	89	(23)	71	(21)
7 to 9	42	(12)	58	(16)	49	(13)	63	(16)	49	(14)
10 or more	19	(5)	26	(7)	29	(8)	29	(8)	37	(11)
Smoking, N (%)										
Never	206	(55)	197	(54)	198	(53)	207	(53)	180	(51)
Ever but not current	84	(22)	81	(22)	85	(23)	79	(20)	73	(21)
Current	85	(23)	90	(24)	94	(25)	102	(26)	98	(28)

Discrepancies in N in some variables are attributable to missing data. Abbreviations BP, blood pressure; bpm, beats per minute.

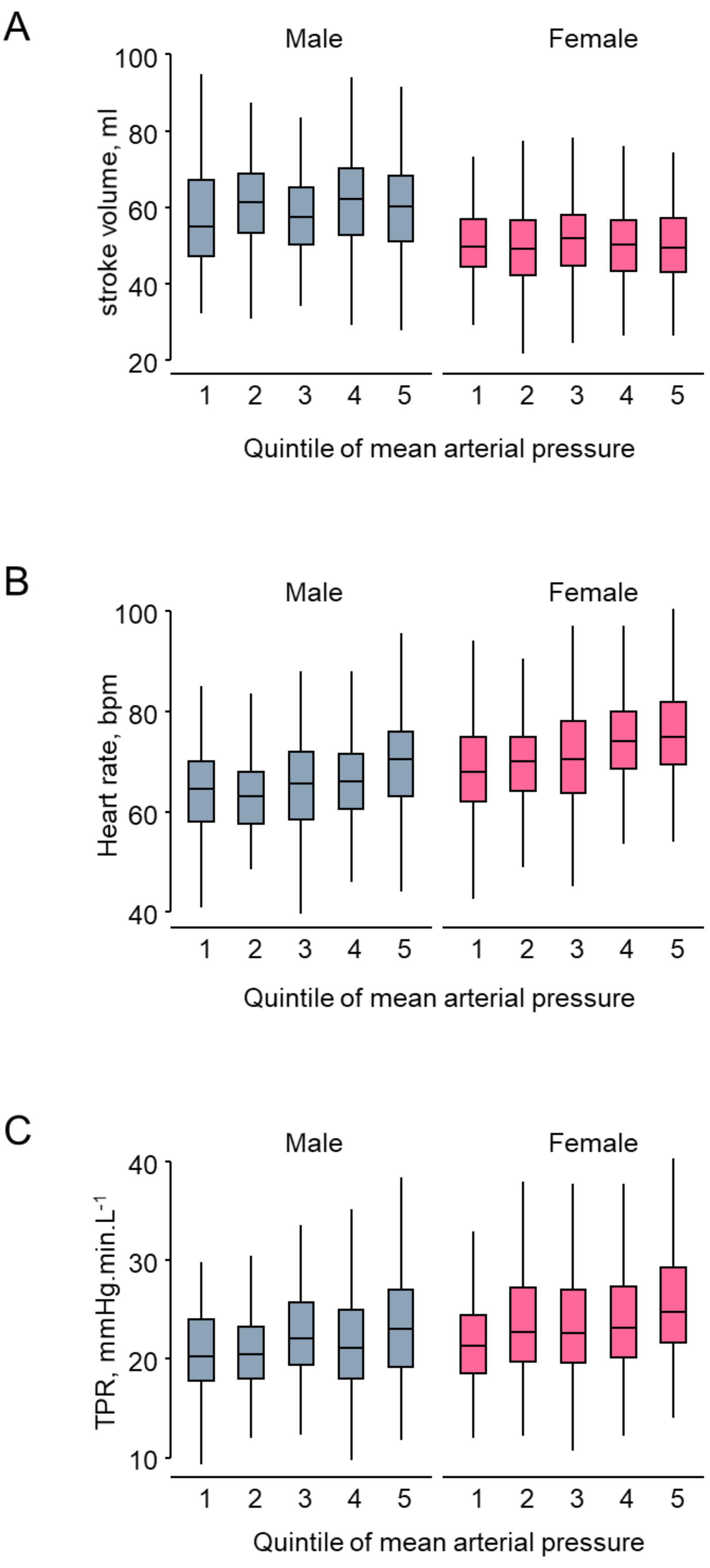

Figure S1. Box and whisker plots of A) stroke volume, B) heart rate C) total peripheral resistance (TPR) by quintiles of mean blood pressure in males and females.
